# Potential for international spread of wild poliovirus via travelers

**DOI:** 10.1186/s12916-015-0363-y

**Published:** 2015-06-04

**Authors:** Annelies Wilder-Smith, Wei-Yee Leong, Luis Fernandez Lopez, Marcos Amaku, Mikkel Quam, Kamran Khan, Eduardo Massad

**Affiliations:** Lee Kong Chian School of Medicine, Nanyang Technological University, Mandalay Road 11, Singapore, 308232 Singapore; Institute of Public Health, University of Heidelberg, Heidelberg, Germany; Florida International University, Miami, USA; Faculty of Veterinary Medicine, University of São Paulo, São Paulo, Brazil; Department of Public Health and Clinical Medicine, Epidemiology and Global Health, Umeå University, Umeå, Sweden; Li Ka Shing Knowledge Institute, St. Michael’s Hospital, Toronto, ON Canada; Faculty of Medicine, Division of Infectious Diseases, University of Toronto, Toronto, ON Canada; School of Medicine, University of São Paulo, São Paulo, Brazil; London School of Hygiene and Tropical Medicine, London, UK

**Keywords:** Poliomyelitis, Travel, Importation, International spread, Secondary cases, Polio vaccination, India, Hajj pilgrimage, Policy, Polio eradication, Mathematical model

## Abstract

**Background:**

The endgame of polio eradication is hampered by the international spread of poliovirus via travelers. In response to ongoing importations of poliovirus into polio-free countries, on 5 May 2014, WHO’s Director-General declared the international spread of wild poliovirus a public health emergency of international concern. Our objective was to develop a mathematical model to estimate the international spread of polio infections.

**Methods:**

Our model took into account polio endemicity in polio-infected countries, population size, polio immunization coverage rates, infectious period, the asymptomatic-to-symptomatic ratio, and also the probability of a traveler being infectious at the time of travel. We applied our model to three scenarios: (1) number of exportations of both symptomatic and asymptomatic polio infections out of currently polio-infected countries, (2) the risk of spread of poliovirus to Saudi Arabia via Hajj pilgrims, and (3) the importation risk of poliovirus into India.

**Results:**

Our model estimated 665 polio exportations (>99 % of which were asymptomatic) from nine polio-infected countries in 2014, of which 78.3 % originated from Pakistan. Our model also estimated 21 importations of poliovirus into Saudi Arabia via Hajj pilgrims and 20 poliovirus infections imported to India in the same year.

**Conclusion:**

The extent of importations of asymptomatic and symptomatic polio infections is substantial. For countries that are vulnerable to polio outbreaks due to poor national polio immunization coverage rates, our newly developed model may help guide policy-makers to decide whether imposing an entry requirement in terms of proof of vaccination against polio would be justified.

## Background

Polio will remain a global problem as long as there is still one case in the world. By 2012, the annual number of polio cases due to wild poliovirus (WPV) had decreased by >99 % since the polio eradication program was launched in 1988. However, three countries never interrupted WPV transmission: Afghanistan, Nigeria, and Pakistan [1]. In 2013 an upsurge of cases was observed caused by outbreaks in five previously polio-free countries, triggered by importation of WPV via travelers. Preventing the further spread of WPV into polio-free countries and the ensuing outbreaks is therefore a top priority in eradicating polio.

Given WPV’s infectiousness and long period of virus excretion, epidemics in previously polio-free countries via travelers importing WPV is not surprising, and has been documented on many occasions. Reinfection of 19 polio-free African countries occurred in 2009 alone [2]. The outbreak of 199 polio cases in Somalia in 2013-2014 was due to an introduction of poliovirus of Nigerian origin [3]. A large outbreak of poliomyelitis, with 463 laboratory-confirmed cases, took place in 2010 in Tajikistan after a single importation of WPV from India in 2009, with further expansion into neighboring Kazakhstan, Russia, Turkmenistan, and Uzbekistan [4]. After being polio-free for more than 10 years, an outbreak occurred in China in 2011 in Xinjiang imported from neighboring Pakistan [5]. In more recent years, the upsurge of polio cases in Pakistan led to exportation of the virus to the Middle East with new alarming outbreaks in Syria and Iraq [6, 7]. Furthermore, a higher proportion of adults has been observed in those affected by the outbreaks as a result of importation [8]. The Hajj pilgrimage has also been considered a high risk for importation of WPV from the remaining polio-infected countries that to a large extent contain Muslim populations [9]. In summary, from 2003 to 2014, there were 191 documented new importation events into previously polio-free countries, resulting in 3,763 reported cases of paralytic polio in 43 countries and costing $1.15 billion in additional funds from international organizations and agencies alone for outbreak control [10].

In response to ongoing importations of poliovirus into polio-free countries, on 5 May 2014, the Director-General of the World Health Organization (WHO) declared the international spread of WPV a public health emergency of international concern (PHEIC). Given that such a declaration has only been made for Ebola and influenza, this declaration highlights how seriously WHO considers the threat to polio eradication due to the ongoing international spread of poliovirus. WHO announced temporary recommendations to ensure that all travelers departing from polio-infected countries receive a polio vaccination (oral or injectable polio vaccine) between 4 weeks and 12 months before international travel, and should ensure that such travelers are provided with proof of vaccination [11]. India did not even wait for this declaration. In early 2014, India was declared polio-free by WHO after a three-year interval without any polio cases [12]. This achievement came at a high price. India is now understandably concerned about reintroduction via travelers - a concern that is justified given its proximity to two countries with currently the highest number of polio cases (Pakistan and Afghanistan). To prevent reintroduction of polio, India therefore decided to tighten cross-border travel rules by introducing a new polio vaccine requirement for all travelers from polio-infected countries entering India. The Government of India’s Ministry of Health and Family Welfare has announced that “Resident nationals of the currently seven polio infected countries are required to receive a dose of oral polio vaccine (OPV), regardless of age and vaccination status, at least four weeks prior to departure to India. A certificate of vaccination with OPV is required for resident nationals of these countries while applying for entry visa to India” [13].

Given WHO’s recent focus on the contribution of the international spread of poliovirus via travelers, it is important and timely to assess the potential for further spread. Reliance on reported events of importation will only underestimate the true importation risk, as not every imported case will be detected and reported, especially in countries with poor surveillance systems. Furthermore, not every imported case will lead to secondary cases, and polio importations without secondary cases are likely to remain unnoticed. Both asymptomatic and symptomatic cases can contribute to transmission [14], and asymptomatic polio infections (that also remain unreported) need to be taken into account when assessing the international spread of polio. In the absence of reliable surveillance data, mathematical modeling is necessary to estimate the number of importations or exportations of polio.

Our objective was to develop a mathematical model to estimate the international spread of symptomatic and asymptomatic polio infections. We then applied our model to three scenarios: (1) number of exportations of both symptomatic and asymptomatic polio infections out of currently polio-infected countries, (2) the risk of spread of poliovirus to Saudi Arabia via Hajj pilgrims, and (3) the importation risk of poliovirus into India via travelers from polio-infected countries.

## Methods

We first obtained data on the number of polio cases reported to WHO in the years 2010 to 2014 [15] including obtaining the number of polio cases classified by WHO as being the result of importation. We calculated the proportion of imported polio over all polio cases per year for the 15 years from 2010 to 2014.

We then adapted a Susceptible -> Infected -> Recovered (SIR) model and fed the following facts and parameters about polio into the model: published estimates of the ratio of inapparent to paralytic illness vary from 50:1 to 1,000:1 (usually 200:1) [14]. We assumed a ratio of 200:1. Persons infected with wild poliovirus (WPV) are most infectious from 7 to 10 days before and after the onset of symptoms, but poliovirus may be present in the stool for up to 3 to 6 weeks [14], with the mean duration of WPV type 1 excretion in fecal specimens being 24 days (median, 20 to 29 days), with a range of 1 to 114 days [16, 17]. We assumed that the duration of infectiousness for our models is 4 weeks and that the life expectancy for every country is equal to 60 years (1/*μ*_*i*_=60). We also assumed, for simplicity, that the probability of poliovirus infection is independent of the probability of travel and that poliovirus infections are homogeneously distributed in polio-affected countries.

In the following paragraphs we explain how the model was developed. The model variables and parameters are described in Table 1. The model equations are shown in Box 1.

**Table 1:** Variables and parameters of the model

Variable	Biological description	Sources
*S*_*i*_(*t*)	Number of susceptible individuals in country *i* at time *t*	Calculated from the total population in country *i* at year *t* times the probability of not being vaccinated
*I*_*i*_(*t*)	Number of polio cases in country *i* at time *t*	Calculated from the model
*p*_*i*_(*t*)	Prevalence of polio in country *i* at time *t*	Calculated from the model
Parameter	Biological description	
*λ*_*i*_(*t*)	Force of infection in country *i* at time *t*	Calculated from the number of reported cases in country *i* at year *t*
1/*μ*_*i*_	Life expectancy of population in country *i*	[26]
1/*γ*_*i*_	Average duration of infection in country *i*	Reference [14]

The general equation describing the time variation in reported polio cases in country _*i*_, in year *t, I*_*i*_*(t)*, is given by Equation (1) in Box 1.

In Equation (1), λ_i_(t) is the force of infection (per capita number of new infections per time unit), *S*_*i*_(*t*) is the number of susceptible individuals at time _*t*_, $\frac{1}{\mu_i}$ is the life expectancy of the population in the country _*i*_, and $\frac{1}{\gamma_i}$ is the average duration of infection in country _*i*_. The number of susceptible individuals is given by the size of the population multiplied by one minus the proportion of polio vaccination coverage. Equation (1) describes the dynamics of infectious individuals. It expresses the time variation in the number of infective people, and it contains two terms, one income (positive) term provided by the disease incidence (with rate λ_i_(t)S_i_(t)) and one outcome (negative) term provided by natural deaths (with rate *μ*_*i*_), and the recovery of infected individuals (with rate *γ*_*i*_).

The number of polio cases in country *i* at year *t* is given by the number of cases at the year the cohort started to be followed up *I(0)* times the number of individuals who survived up until year *t,* plus the number of new cases of infection (see Equation (2) in Box 1).

We consider the reported number of cases for the year _*t*_ in country _*i*_ (Equation (2) of Box 1) and assumed that this annual incidence is constant along each year, that is, there is no seasonal variation.

As we assumed a 200:1 asymptomatic-to-symptomatic ratio, we multiplied the number of reported cases at year _*t*_ in country *i* (*Λ*_*i*_(*t*) in Equation (3) of Box 1).

Dividing Equation (3) from Box 1 by the number of susceptible individuals *S*_*i*_(*t*) at year *t* in country *i*, we obtain the prevalence *p*_*i*_(*t*) in year *t* in country *i*. Multiplying *p*_*i*_(*t*) by the number of non-immune travelers from country _*i*_, we obtain the expected number of polio cases among travelers.

We conducted a sensitivity analysis for the model, which is described in detail in Box 2. After developing the model, we applied it to three scenarios:

1. **Exportation of polio infections (asymptomatic and symptomatic) from polio-infected countries in the years 2013-2014.**
  We obtained data on the countries that had polio cases reported to WHO from the website indicated in Ref. [15]. The number of travelers departing these polio-affected countries was obtained from the International Air Transport Association (IATA). We applied Equation (3) to estimate the expected number of polio exportations, taking into account the degree of polio immunization coverage rates (oral polio vaccine) for the countries, outbound travel, asymptomatic-symptomatic ratio, and population size.
2. **International spread of poliovirus via Hajj pilgrims in the years 2013 and 2014.**
  As the Ministry of Health of Saudi Arabia has withdrawn its public website on the number of annual Hajj pilgrims, we estimated the number of Hajj pilgrims from earlier reports in the published literature [18]. We then applied the same equation as for scenario 1 to estimate the risk of international importation of poliovirus via Hajj pilgrims into Saudi Arabia.
3. **Importation of poliovirus into India in the years 2010-2014:**
  We selected the seven countries for which India has issued the requirement for proof of vaccination against polio (Afghanistan, Ethiopia, Kenya, Nigeria, Pakistan, Somalia, and Syria) [13] and obtained the numbers of nationals from these countries traveling to India from India’s tourism statistics [19]. The travelers include those arriving by air, ground, and sea. The population size per country was obtained from the World Bank. The number of wild-type polio cases and the national polio immunization coverage rates were obtained from WHO [20]. We also obtained data on travel volume and force of infection for the years 2010 to 2014, using the same assumptions for all the variables except for population size, travel volume, and force of infection, where we used the yearly available data. We also took into account that in different years additional polio-infected countries existed.

## Results

**Fig. 1:**
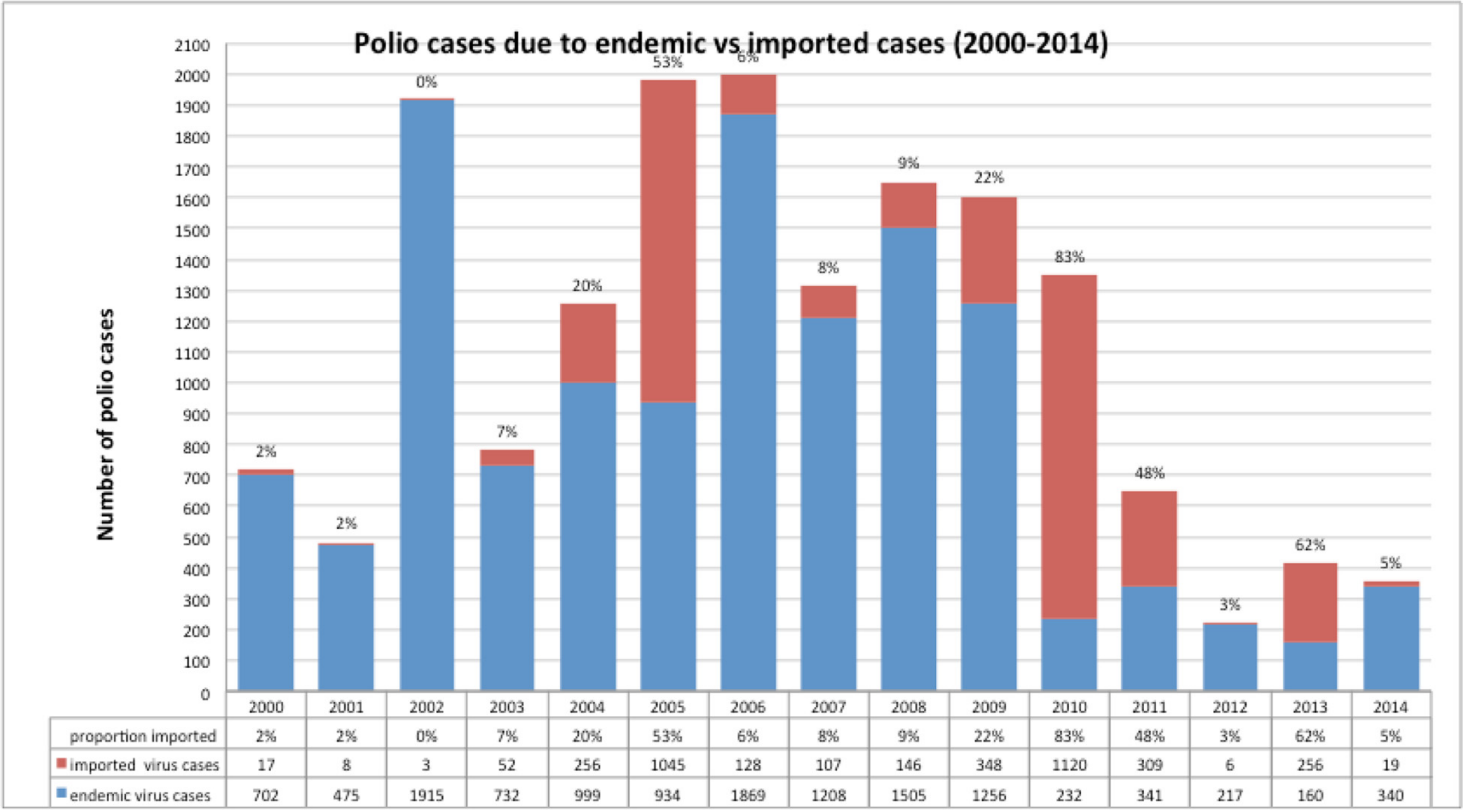
Polio cases (endemic versus imported) 2000-2014

In 2013, imported polio cases accounted for the majority of all reported polio cases 256 of 416 cases (corresponding to Fig. 1) shows the proportion of imported polio cases per year from 2000-2014. Over those 15 years, the proportion of polio cases as a result of importation increased significantly, and accounted for 62 % in the year 2013. The years 2005 and 2010 were also the years that showed a very high proportion of polio as a result of importation (53 % and 83 %, respectively).

In Table 2, we summarize the number of modeled exported cases from polio-infected countries in the years 2013 and 2014. The expected number of exported polio cases in 2013 was 462, of which half were contributed by Pakistan (n = 158) and Somalia (n = 121). Among these countries, Somalia had the highest number of polio cases in 2013 (n = 194) in that particular year and the lowest vaccination coverage rate (47 %). In the year 2014, Somalia had far fewer endemic polio cases, but polio infections tripled in Pakistan. Given the high travel volume between Pakistan and India, the estimated overall annual polio exportations increased from 2013 to 2014 to 665.

**Table 2:** Estimated exported polio infections from polio-infected countries for 2013 and 2014, accounting for a ratio of apparent to inapparent polio infections of 200:1

Country	Outbound travelers ^a^ (2012)	Vaccination coverage (2013) ^b^	Population size (2013) ^c^	Symptomatic cases (2013) ^d^	Symptomatic cases (2014) ^d^	Asymptomatic infections (2013)	Asymptomatic infections (2014)	Exported polio infections (2013)	Exported polio infections (2014)
Pakistan	4,655,292	0.72	182,142,594	93	306	18,600	61,200	158	521
Afghanistan	792,905	0.71	30,551,674	14	28	2,800	5,600	24	48
Nigeria	1,829,727	0.67	173,615,345	53	6	10,600	1,200	37	4
Somalia	98,390	0.47	10,495,583	194	5	38,800	1,000	121	3
Syria	629,804	0.52	22,850,000	35	1	7,000	200	64	2
Ethiopia	1,249,894	0.7	94,100,756	9	1	1,800	200	8	1
Kenya	2,028,017	0.82	44,353,691	14	0	2,800	NC	43	NC
Cameroon	505,550	0.88	21,699,631	4	5	800	1,000	6	8
Equatorial Guinea	168,530	0.30	757,014	0	5	NC	1,000	NC	74
Iraq	1,033,884	0.70	33,417,476	0	2	NC	400	NC	4
Total	12,991,993		613,983,764	416	359	83,200	71,800	462	665

^a^ Data from International Air Transport Association (source: Bluedot; Kamran Khan)

^b^ [27]

^c^ [28] (2013)

^d^ [15]

NC = not calculated

Table 3 shows the estimated number of exported cases by outbound travel of Hajj pilgrims from the countries that reported polio cases to WHO in 2013 and 2014. Our findings indicated that in 2013 a total of 20 cases were exported from these polio-infected countries. Somalia (n = 8) and Pakistan (n = 6) were the two countries with the highest exportation, contributing 70 % of the total exported cases. Although there were reported polio cases in Ethiopia, Kenya, and Cameroon, based on our model, they did not contribute to any exportation. In 2014, the number of importations via Hajj pilgrims was 21, and Pakistan contributed to most of these importations (19 out of 21).

**Table 3:** Estimated imported polio infections to Saudi Arabia during the Hajj pilgrimage from polio-infected countries in 2013 and 2014

Country	Hajj pilgrims^a^ (2012)	Vaccination coverage (2013) ^b^	Population size (2013) ^c^	Symptomatic cases (2013)^d^	Symptomatic cases (2014) ^d^	Asymptomatic infections (2013)	Asymptomatic infections (2014)	Imported polio infections (2013)	Imported polio infections (2014)
Pakistan	172,610	0.72	182,142,594	93	306	18,600	61,200	6	19
Afghanistan	33,011	0.71	30,551,674	14	28	2,800	5,600	1	2
Nigeria	98,559	0.67	173,615,345	53	6	10,600	1,200	2	0
Somalia	6,540	0.47	10,495,583	194	5	38,800	1,000	8	0
Syria	30,921	0.52	22,850,000	35	1	7,000	200	3	0
Ethiopia	4,009	0.7	94,100,756	9	1	1,800	200	0	0
Kenya	2,480	0.82	44,353,691	14	0	2,800	NC	0	NC
Cameroon	3,570	0.88	21,699,631	4	5	800	1,000	0	0
Equatorial Guinea	NA	0.30	757,014	0	5	NC	1,000	NC	NC
Iraq	35,748	0.70	33,417,476	0	2	NC	400	NC	0
Total	384,968		613,983,764	416	359	83,200	71,800	20	21

^a^ [18]

^b^ [27]

^c^ [28]

^d^ [15]

NA = not available

NC = not calculated

**Fig. 2:**
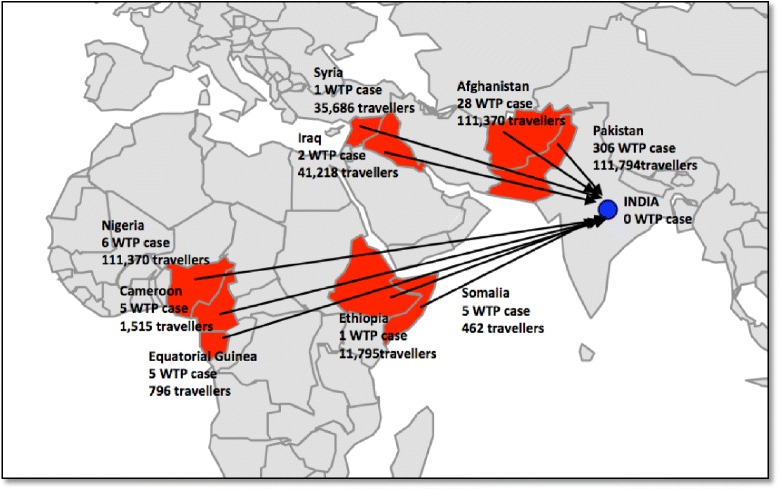
Travelers from polio-infected countries entering India in 2014

Figure 2 shows the routes of potential polio importation into India in 2014. The results of the application of Equation (3) for the case of polio importation into India by travelers are shown in Tables 4 and 5. The total number of estimated polio importations from nine polio-infected countries summed up to 20 polio infections imported to India in the year 2014 (Table 4). Afghanistan and Pakistan, both countries in close geographical proximity to India, contributed all 20 of the imported polio infections (13 from Pakistan, 7 from Afghanistan). Table 5 shows that the highest importation risk (24 estimated importations) into India for the years 2010-2013 was in the year 2010 when 20 countries had at least one polio case. In 2011, there were 22 estimated importations; in 2012, 11 importations; and in 2013, 13 importations of polio infections into India.

**Table 4:** Estimated importations of polio infections into India in the year 2014

Country	Travelers to India	Polio cases (2014)^a^	Asymptomatic polio	Vaccination coverage (2013) ^b^	Population size (2013) ^c^	Force of infection	p(t)	Importation
Pakistan	111,794 (2013)^d^	306	61,200	0.72	182,142,594	0.000336	0.000111844	13
Afghanistan	111,370 (2013)^d^	28	5,600	0.71	30,551,674	0.000183296	6.10136E-05	7
Nigeria	65,968 (2012)^e^	6	1,200	0.67	173,615,345	6.91183E-06	2.30073E-06	0
Somalia	462 (2012)^e^	5	1,000	0.47	10,495,583	9.52782E-05	3.17152E-05	0
Syria	35,686 (2012)^e^	1	200	0.52	22,850,000	8.75274E-06	2.91351E-06	0
Ethiopia	11,795 (2012)^e^	1	200	0.7	94,100,756	2.12538E-06	7.07474E-07	0
Cameroon	1,515 (2012)^e^	5	1,000	0.88	21,699,631	4.60837E-05	1.53398E-05	0
Equatorial Guinea	796 (2012)^e^	5	1,000	0.30	757,014	0.00132098	0.000439713	0
Iraq	41,218 (2013) ^d^	2	400	0.70	33,417,476	1.19698E-05	3.98437E-06	0
Total	380,604	359	71,800		569,630,073			20

^a^ [15]

^b^ [27]

^c^ [28]

^d^ [29]

^e^ Data from United Nations World Tourism Organization

**Table 5:** Estimated importations of polio infections into India (2010-2013)

Country	Travelers to India^a^	Polio cases^d^	Asymptomatic polio	Vaccination coverage^e^	Population size^f^	Importation
2010						
Pakistan	51,749	144	28,800	0.82	173,149,306	3
Nigeria	23,893	21	4,200	0.54	159,707,780	0
Afghanistan	73,389	25	5,000	0.66	28,397,812	4
Niger	475	2	400	0.75	15,893,746	0
Chad	212	26	5,200	0.43	11,720,781	0
DRC	NA	100	20,000	0.76	62,191,161	NC
Angola	1,620	33	6,600	0.92	19,549,124	0
Mali	495	4	800	0.8	13,985,961	0
Congo	1,930	441	88,200	0.9	4,111,715	14
India	NA	42	8,400	0.7	1,205,624,648	NC
Uganda	3,011	4	800	0.79	33,987,213	0
Russia	122,048	14	2,800	0.98	142,385,523	1
Liberia	177	2	400	0.71	3,957,990	0
Nepal	104,374	6	1,200	0.83	26,846,016	2
Kazakhstan	8,786	1	200	0.98	16,321,581	0
Tajikistan	NA	460	92,000	0.95	7,627,326	NC
Turkmenistan	NA	3	600	0.96	5,041,995	NC
Senegal	NA	18	3,600	0.79	12,950,564	NC
Mauritania	131	5	1,000	0.52	3,609,420	0
Sierra Leone	NA	1	200	0.84	5,751,976	NC
TOTAL	392,290	1,352	270,400		1,952,811,638	24
2011						
Pakistan	48,640	198	39,600	0.75	176,166,353	4
Nigeria	33,537	62	12,400	0.49	164,192,925	1
Afghanistan	89,605	80	16,000	0.68	29,105,480	16
Kenya	30,045	1	200	0.88	42,027,891	0
Niger	600	5	1,000	0.44	16,511,462	0
Chad	1,198	132	26,400	0.4	12,080,037	1
DRC	NA	93	18,600	0.77	63,931,512	NC
CAR	37	4	800	0.47	4,436,217	0
China	142,218	21	4,200	0.99	1,344,130,000	0
Guinea	341	3	600	0.63	11,161,530	0
Cote d’Ivoire	637	36	7,200	0.58	19,389,954	0
Angola	1,891	5	1,000	0.85	20,180,490	0
Mali	815	7	1,400	0.78	14,416,737	0
Congo	3,623	1	200	0.9	4,225,359	0
Gabon	226	1	200	0.75	1,594,034	0
India	NA	1	200	0.7	1,221,156,319	NC
TOTAL	353,413	650	130,000		3,144,706,300	22
2012						
Pakistan	58,846	58	11,600	0.72	179,160,111	1
Nigeria	36,762	122	24,400	0.52	168,833,776	2
Afghanistan	95,231	37	7,400	0.71	29,824,536	8
Niger	497	1	200	0.78	17,157,042	0
Chad	194	5	1,000	0.56	12,448,175	0
TOTAL	191,530	223	44,600		407,423,640	11
2013						
Pakistan	111,794^c^	93	18,600	0.72	182,142,594	4
Afghanistan	111,370^c^	14	2,800	0.71	30,551,674	3
Nigeria	34,522^b^	53	10,600	0.67	173,615,345	1
Somalia	462^b^	194	38,800	0.47	10,495,583	1
Syria	35,686^b^	35	7,000	0.52	22,850,000	4
Ethiopia	18,106^b^	9	1,800	0.7	94,100,756	0
Kenya	40,484^c^	14	2,800	0.82	44,353,691	1
Cameroon	1,515^b^	4	800	0.88	21,699,631	0
TOTAL	353,939	416	83,200		579,809,274	13

^a^ [30]

^b^ Data from United Nations World Tourism Organization

^c^ [29]

^d^ [31]

^e^ [27]

^f^ [28]

NA = not available

NC = not calculated

Sensitivity analysis: The sensitivity analysis in Box 2 shows that the model is about 12 times less sensitive to *μ* and *γ* than to *λ*. In other words, the force of infection in the epidemic source country is the main driving factor for the importation risk, combined with the travel volume. However, note that *λ* is covariate with *μ* and *γ*. As we do not know the exact relationship among them, it is not possible to calculate either $\frac{\partial \lambda }{\partial \mu }$ or $\frac{\partial \lambda }{\partial \gamma }$, and thus the sensitivity analysis incomplete.

## Discussion

Although the number of global polio cases has been declining over the past 15 years, as shown in Fig. 1, the proportion of polio as a result of importation is increasing, which is a major concern to polio eradication. The proportion has ranged from 0 to 62 % over the past 15 years, with the years 2005, 2010, and 2013 reporting the highest proportions. However, these numbers do not reflect importation events, but rather the overall polio cases in non-endemic polio countries as a result of importation from polio-endemic or polio-infected countries (Pakistan, Afghanistan, and Nigeria being the only endemic countries since 2010). These proportions hence cannot inform from where the importation originated and how many travelers imported polio. Here, we have developed a mathematical model to address this gap. Our model not only takes into account polio endemicity (force of infection), population size, polio vaccination coverage, and infectious period, but also the probability of a traveler being infectious at the time of travel. As both symptomatic and asymptomatic polio infections are known to transmit the virus, our model includes asymptomatic cases. As the ratio of asymptomatic to symptomatic polio is very high, most of our estimated importations of poliovirus are via asymptomatic travelers, and hence our estimated numbers of importation/exportation appear high. Our sensitivity analysis shows that the force of infection in the epidemic source country is the main driving factor for the importation risk, combined with the travel volume.

In the year 2014, out of nine polio-affected countries, we modeled that 665 polio infections (of which 662 were asymptomatic, and only 3 symptomatic) were exported globally. The majority of these poliovirus exportations originated from Pakistan (521 out of 665; 78.3 %). The number of polio exportations will vary from year to year. In 2013, for example, there were 462 estimated exportations of polio infections, and less than half can be attributed to Pakistan, with Somalia contributing almost the same amount as Pakistan, as there was a major polio outbreak in Somalia that year (Table 2). As per the sensitivity analysis, we found that for every 1 % variation in the force of infection there will be approximately 1 % variation in the time-dependent prevalence in the country.

About 600 potential poliovirus importations from nine polio-infected countries in one year into any country in the world present a sizable problem. However, our model cannot tell us how many of these importation events will lead to secondary cases or even outbreaks. It is the national coverage of polio immunization in a country that will determine its vulnerability for polio outbreaks after importation. The polio vaccination coverage rate in any given country together with the quality of its surveillance system and the speed of outbreak response will determine the magnitude of outbreaks following importation. Countries with high polio vaccination coverage rates may still see importations of polio cases (the majority of which will be asymptomatic and hence not detected), but are unlikely to see secondary cases. For example, countries such as Australia, New Zealand, the United States, and Singapore have reported importations, but given their high immunization coverage rates, no single secondary cases occurred [21, 22]. For Africa, Andre Mach *et al.* recently assessed the risk for polio outbreaks for 2013-2014, and the authors found 15 countries to be at high risk for WPV outbreaks, 5 at moderate-to-high risk, and 6 at low risk [23]. In 15 of the 33 African countries, less than half of the population resides in areas where surveillance performance indicators have met only minimum targets [23, 24].

Every year, the Hajj pilgrimage brings more than 2 million pilgrims from all over the world to Saudi Arabia, an event that is characterized by overcrowding [9]. Many of these pilgrims are from Nigeria, Pakistan, and Afghanistan - the three remaining polio-endemic countries - and other predominantly Moslem countries that are polio-infected. Hence, there has been long-standing concern about the potential introduction of polio into the Hajj with subsequent international spread. Our model estimated 20 importations of poliovirus into Saudi Arabia via Hajj pilgrims in the year 2013, and 21 in the year 2014. About 20 potential importations need to be taken seriously. Indeed, the Kingdom of Saudi Arabia has introduced mandatory polio vaccination at the point of entry for all pilgrims coming from polio-infected countries [9].

To prevent reintroduction of the poliovirus, in January 2014 India decided to tighten cross-border travel rules by introducing a new polio vaccine requirement for all travelers from polio-infected countries entering India [12, 25]. We previously estimated the number of travelers who would be affected by this new rule imposed by the Indian government to be approximately 233,800 travelers annually from the seven countries to India, and 346,800 Indian national residents to these seven countries, in the year 2013 [25]. Our model now adds more information: our findings estimate that 13 polio importations occurred in the year 2013, and 20 in 2014. In 2014, 100 % of these polio importations into India originated from Pakistan and Afghanistan, which is not surprising given that these two countries carry the main remaining burden of polio and have the highest travel volume to India. Although the travel volume from Pakistan to India was of the same order of magnitude as that for Afghanistan to India, the force of infection of polio was higher in Pakistan, and hence the probability of importing polio from Pakistan to India is higher compared to Afghanistan. Given the high asymptomatic-to-symptomatic ratio, most likely all 20 imported infections were asymptomatic. The total expected number of importations may seem low at first sight. However, one must consider that for the year 2014 this figure implies an incidence of 20 per 380,604 travelers (5 polio infections per 100,000 travelers), which is far from negligible. In other words, of the total number of 72,159 asymptomatic and symptomatic polio infections in the nine countries, 20 (about 0.027 %) of them were estimated to have traveled to India at the time of polio infection associated with viral shedding. The number of secondary cases will depend on the polio immunization coverage rate, which according to WHO was 70 % for India [20]. Fortunately, no single imported polio case was detected or officially reported in India in the years 2013 or 2014. We added an analysis of the preceding years (2010–2012) for comparison. The years 2010 and 2011 saw high numbers of importations mainly due to the much higher number of countries that reported at least one case of polio. As even one importation event can lead to substantial outbreaks associated with hundreds of million dollars spent for their control [3, 4], our finding of 20 importations in one year (2014) lends support to India’s strategy to protect its country from reinfection via travelers. India’s new vaccine policy would affect approximately 380,000 travelers, and approximately 19,000 vaccinations need to be administered per one averted poliovirus importation. Given the tragic consequences of re-infecting India with polio, the number of travelers that need to be vaccinated to avert poliovirus importation would be justified. India’s emphasis should be on preventing polio importation from Pakistan and Afghanistan.

Our model had the following limitations. For lack of data, we assumed that the probability of poliovirus infection is independent of the probability of travel; and for simplicity, we assumed that poliovirus infections are homogeneously distributed in polio-affected countries. These assumptions will lead to an overestimate of our results. For the estimation of outbound travel from polio-affected countries (scenario 1, Table 2), we obtained data from IATA. These data capture an estimated 90 % of all passengers traveling on commercial airlines worldwide, but do not incorporate land travel. Hence, they tend to underestimate international travel volumes between countries that share contiguous land borders (that is, where land-based border crossings are common). The true exportation numbers may therefore be even higher than we estimated, if ground travel volume is included. Unfortunately, data on ground travel volume is difficult to obtain for many of the polio-affected countries. However, India does publish the number of travelers into its country, and these data include air and land travel. As there are a lot of border crossings via land from Pakistan to India, it was important to include such land travel data for estimating poliovirus importation into India (scenario 3, Tables 4 and 5).

## Conclusions

As long as polio exists anywhere in the world, it can be imported anywhere. Our model can be applied to all countries and provides an additional tool to estimate the risk of importation based on the main contributing factors such as travel volume and polio endemicity. In particular, for countries that are vulnerable to polio outbreaks due to poor national polio immunization coverage rates, our model may help guide policy-makers to decide whether imposing an entry requirement in terms of proof of vaccination against polio would be justified.

## Box 1. Model equations as explained in the main text

The general equation for the variation in polio cases in country *i*, in year *t, I*_*i*_(*t*), is given by:

1 $$ \frac{d{I}_i(t)}{dt}={\lambda}_i(t){S}_i(t)-\left({\mu}_i+{\gamma}_i\right){I}_i(t) $$

where *λ*_*i*_(*t*) is the force of infection (per capita number of new infections per time), *S*_*i*_(*t*) is the number of susceptible individuals at time _*t*_, $\frac{1}{\mu_i}$ is the life expectancy of the population in country _*i*_, and $\frac{1}{\gamma_i}$ is the average duration of infection in country *i*. The number of susceptible individuals is given by the size of the population multiplied by one minus the proportion of polio vaccination coverage. Equation (1) describes the dynamics of infectious individuals. It expresses the time variation in the number of infective people and it contains two terms, one income (positive) term provided by the disease incidence (with rate *λ*_*i*_(*t*)*S*_*i*_(*t*)) and one outcome (negative) term provided by natural deaths (with rate *μ*_i_), and the recovery of infected individuals (with rate *γ*_*i*_).

Equation (1) can be integrated by standard methods, resulting in:

2 $$ {I}_i(t)={I}_i(0){e}^{-\left({\mu}_i+{\gamma}_i\right)t}+{\displaystyle \underset{0}{\overset{t}{\int }}}{\lambda}_i\left({t}^{\prime}\right){S}_i\left({t}^{\prime}\right){e}^{-\left({\mu}_i+{\gamma}_i\right)\left(t-{t}^{\prime}\right)}dt $$

We consider ${\displaystyle \underset{0}{\overset{t}{\int }}}{\lambda}_i\left({t}^{\prime}\right){S}_i\left({t}^{\prime}\right)dt$, the reported number of cases along the year *t* in country *i*, and assume this annual incidence as constant in each year. Equation (2), therefore, simplifies to

3 $$ {I}_i(t)={I}_i(0){e}^{-\left({\mu}_i+{\gamma}_i\right)t}+{\varLambda}_i(t)\left\{\frac{\left[1-{e}^{-\left({\mu}_i+{\gamma}_i\right)t}\right]}{\left({\mu}_i+{\gamma}_i\right)}\right\} $$

Where ${\varLambda}_i(t)={\displaystyle \underset{0}{\overset{t}{\int }}}{\lambda}_i\left({t}^{\prime}\right){S}_i\left({t}^{\prime}\right)dt$ for each individual year *t*.

Dividing Equation (3) by the number of susceptible individuals *S*_*i*_(*t*) in year *t* in country *i*, we obtain the prevalence *p*_*i*_(*t*) in year *t* in country *i*:

4 $$ {p}_i(t)={p}_i(0){e}^{-\left({\mu}_i+{\gamma}_i\right)t}+{\varLambda}_i(t)\left\{\frac{\left[1-{e}^{-\left({\mu}_i+{\gamma}_i\right)t}\right]}{S_i(t)\left({\mu}_i+{\gamma}_i\right)}\right\} $$

Multiplying *p*_*i*_(*t*) by the number of non-immune travelers from country *i*, we obtain the expected number of polio cases among travelers.

## Box 2. Sensitivity of the model to the parameters

For a small variation of parameter *Par*_*i*_, Δ*Par*_*i*_, the variation in the parameter-dependent variable π, Δπ, is given by the well-known error propagation formula^26^:

5 $$ \varDelta \pi ={\displaystyle \sum_i\frac{\partial \pi }{\partial Pa{r}_i}\times \varDelta Pa{r}_i} $$

The relative variation in the risk π, Δπ/π, as a function of the relative variation in the parameters Δ*Par*_*i*_/*Par*_*i*_, is therefore:

6 $$ \frac{\varDelta \pi }{\pi }={\displaystyle \sum_i Pa{r}_i\frac{\partial \pi }{\partial Pa{r}_i}\times \frac{\varDelta Pa{r}_i}{Pa{r}_i}\times \frac{1}{\pi }} $$

We calculated the sensitivity of Equation (3) of Box 1 to the natural mortality rate of hosts, *μ*:

7 $$ \frac{\varDelta p(t)}{p(t)}=\frac{\partial p(t)}{\partial \mu}\times \frac{\varDelta \mu }{\mu}\times \frac{\mu }{p(t)} $$

Where

8 $$ \frac{\partial p(t)}{\partial \mu }=-t{p}_i(0){e}^{-\left({\mu}_2+{\gamma}_i\right)t}-\left\{\left[{\displaystyle \underset{0}{\overset{t}{\int }}}{\lambda}_i\left({t}^{\prime}\right){S}_i\left({t}^{\prime}\right)d{t}^{\prime}\right]\left\{\frac{\left[1-{e}^{-\left({\mu}_i+{\gamma}_i\right)t}\right]}{S_i(t){\left({\mu}_i+{\gamma}_i\right)}^2}\right\}+\left[{\displaystyle \underset{0}{\overset{t}{\int }}}{\lambda}_i\left({t}^{\prime}\right){S}_i\left({t}^{\prime}\right)d{t}^{\prime}\right]\left\{\frac{\left[{e}^{-\left({\mu}_i+{\gamma}_i\right)t}\right]}{S_i(t)}\right\}\right\}\frac{\partial \lambda }{\partial \gamma } $$

is the partial derivative of time-dependent prevalence with respect to the natural mortality rate of hosts *μ*.

An identical result is obtained for $\frac{\partial p(t)}{\partial \gamma }$.

The result of $\frac{\partial p(t)}{\partial \gamma }$ is much simpler:

9 $$ \frac{\partial p(t)}{\partial \lambda }=\frac{\left[1- \exp \left(-\left(\mu +\gamma \right)t\right)\right]}{S(t)\left(\mu +\gamma \right)} $$

Therefore, the equations for the sensitivity analysis are:

10 $$ \begin{array}{c}\hfill \frac{\varDelta p(t)}{\mu }=-t{p}_i(0){e}^{-\left({\mu}_i+{\gamma}_i\right)t}-\left\{\left[{\displaystyle \underset{0}{\overset{t}{\int }}}{\lambda}_i\left({t}^{\prime}\right){S}_i\left({t}^{\prime}\right)d{t}^{\prime}\right]\left\{\frac{\left[1-{e}^{-\left({\mu}_i+{\gamma}_i\right)t}\right]}{S_i(t){\left({\mu}_i+{\gamma}_i\right)}^2}\right\}+\left[{\displaystyle \underset{0}{\overset{t}{\int }}}{\lambda}_i\left({t}^{\prime}\right){S}_i\left({t}^{\prime}\right)d{t}^{\prime}\right]\left\{\frac{\left[{e}^{-\left({\mu}_i+{\gamma}_i\right)t}\right]}{S_i(t)}\right\}\right\}\hfill \\ {}\hfill \frac{\partial \lambda }{\partial \mu}\times \left\{\frac{\varDelta \mu }{\mu}\times \frac{p(t)}{\pi}\right\}\hfill \end{array} $$

11 $$ \begin{array}{l}\frac{\varDelta p(t)}{\gamma }=-t{p}_i(0){e}^{-\left({\mu}_i+{\gamma}_i\right)t}-\left\{\left[{\displaystyle \underset{0}{\overset{t}{\int }}}{\lambda}_i\left({t}^{\prime}\right){S}_i\left({t}^{\prime}\right)d{t}^{\prime}\right]\left\{\frac{\left[1-{e}^{-\left({\mu}_i+{\gamma}_i\right)t}\right]}{S_i(t){\left({\mu}_i+{\gamma}_i\right)}^2}\right\}+\left[{\displaystyle \underset{0}{\overset{t}{\int }}}{\lambda}_i\left({t}^{\prime}\right){S}_i\left({t}^{\prime}\right)d{t}^{\prime}\right]\left\{\frac{\left[{e}^{-\left({\mu}_i+{\gamma}_i\right)t}\right]}{S_i(t)}\right\}\right\}\\ {}\frac{\partial \lambda }{\partial \mu}\times \left\{\frac{\varDelta \mu }{\gamma}\times \frac{p(t)}{\gamma}\right\}\end{array} $$

12 $$ \frac{\varDelta p(t)}{\lambda }=\frac{\left[1- \exp \left(-\left(\mu +\gamma \right)t\right)\right]}{S(t)\left(\mu +\gamma \right)}\times \frac{\varDelta \mu }{\lambda}\times \frac{p(t)}{\lambda } $$

As the sensitivity of the prevalence to the parameters is an explicit function of time, as time passes (*t* → ∞), the values settle to:

13 $$ \frac{\varDelta p}{\lambda}\cong \frac{\varDelta \mu }{\lambda } $$

that is, for every 1 % variation in the force of infection there will be approximately 1 % variation in the time-dependent prevalence.

For the other two parameters *μ* and *γ* the results are:

14 $$ \frac{\varDelta p}{\mu}\cong \frac{1}{12}\frac{\varDelta \mu }{\mu } $$

And

15 $$ \frac{\varDelta p}{\gamma}\cong -\frac{1}{12}\frac{\varDelta \gamma }{\gamma } $$

that is, the model is about 12 times less sensitive to *μ* and *γ* than to *λ*.

## Abbreviations

IATA: International Air Transport Association
OPV: Oral polio vaccine
PHEIC: Public health emergency of international concern
WHO: World Health Organization
WPV: Wild poliovirus
